# Dysbiosis of the Gut Microbiome is associated with Tumor Biomarkers in Lung Cancer

**DOI:** 10.7150/ijbs.35980

**Published:** 2019-09-07

**Authors:** Fang Liu, Jingjing Li, Yubin Guan, Yanfeng Lou, Huiying Chen, Mingyu Xu, Dequan Deng, Jun Chen, Beibei Ni, Lan Zhao, Hongwei Li, Hong Sang, Xiangsheng Cai

**Affiliations:** 1 Nanjing School of Clinical Medicine, Southern Medical University, Jinling Hospital, Nanjing, 210002, People's Republic of China.; 2 Institute of Biotherapy, Southern Medical University, Guangzhou, 510515, People's Republic of China.; 3 Clinical Laboratory, The First Affiliated Hospital of Guangdong Pharmaceutical University, Guangzhou, 510080, People's Republic of China.; 4 Cell-gene Therapy Translational Medicine Research Center, The Third Affiliated Hospital of Sun Yat-sen University, Guangzhou, 510630, People's Republic of China.; 5 Technology center, Guangdong Vitalife Bio-tech Co.,Ltd., Foshan, 528200, People's Republic of China.

**Keywords:** Lung cancer, Gut microbiome, Cytokeratin 19 fragment, Neuron specific enolase, Carcinoembryonic antigen, Fecal, 16SrRNAsequencing

## Abstract

Lung cancer is a malignancy with high morbidity and mortality worldwide. More evidences indicated that gut microbiome plays an important role in the carcinogenesis and progression of cancers by metabolism, inflammation and immune response. However, the study about the characterizations of gut microbiome in lung cancer is limited. In this study, the fecal samples were collected from 16 healthy individuals and 30 lung cancer patients who were divided into 3 groups based on different tumor biomarkers (cytokeratin 19 fragment, neuron specific enolase and carcinoembryonic antigen, respectively) and were analyzed using 16S rRNA gene amplicon sequencing. Each lung cancer group has characterized gut microbial community and presents an elimination, low-density, and loss of bacterial diversity microbial ecosystem compared to that of the healthy control. The microbiome structures in family and genera levels are more complex and significantly varied from each group presenting more different and special pathogen microbiome such as *Enterobacteri*aceae, *Streptococcus*,* Prevotell*a, *etc* and fewer probiotic genera including *Blautia, Coprococcus, Bifidobacterium* and *Lachnospiraceae*. The Kyoto Encyclopedia of Genes and Genomes (KEGG) and COG annotation demonstrated decreased abundance of some dominant metabolism-related pathways in the lung cancer. This study explores for the first time the features of gut microbiome in lung cancer patients and may provide new insight into the pathogenesis of lung cancer system, with the implication that gut microbiota may serve as a microbial marker and contribute to the derived metabolites, development and differentiation in lung cancer system.

## Introduction

Lung cancer is a leading cause of death by cancer worldwide, causing up to 23.1% deaths of all cancers in 2018 according to the WHO. The gut microbiome is recognized as 'second genome' of humans and has attracted considerable attention in recent decades. It is estimated that the gut microbiome contains more than 100 times the genes in the human genome and performs key functions relevant for human health 1. The microbiome participates in nutrient metabolism, vitamin synthesis, pathogenic growth inhibition, maturation and maintenance of immunological system, angiogenesis stimulation and fat storage regulation to ensure a balance or homeostasis in the body 2, 3. Based on the complex functions of gut microbiome in altering the immune system, recently published studies confirmed a link of gut dysbiosis in some malignancies 4, 5. For example, gut microbiome could trigger systemic autoimmune disease by skewing dual TCR expression in the host and modulate hepatic natural killer T (NKT) cells levels to indirectly control liver tumor growth by metabolizing bile acids in mice 6. Moreover, the impact of gut microbiome disruption by antibiotics potentially impair immunological response resulted in low-density, diversified microbial ecosystem in cancers, which further imply the potential pathogenesis of gut microbiota in cancer host and could determine the efficacy of immunotherapy 7. For example, the gut microbiome colonization of vancomycin-treated mice with clostridium scindens or feeding secondary bile acids to vancomycin-treated mice resulted in a decrease in hepatic NKT cells and more liver metastases 8. Patients treated with antibiotics for routine indications shortly before, during or after treatment with anti-PD-1/PD-L1 mAB had significantly lower progression-free survival and overall survival rates compared with patients who had not received antibiotics 9. However, lung cancer relevant studies were limited thus a strong interest emerged in characterizing the role of gut microbiota in lung cancer.

Meanwhile, the advancement in respiratory immune system further intensifies our interest in the interaction between lung cancer and gut microbiome 10. In this system, the concept of gut-lung axis was proposed and gut microbiota was confirmed to be involved in the pathogenesis of common lung diseases such as asthma, chronic obstructive pulmonary disease (COPD) and respiratory infections asymptomatic colonization 10-12. These published studies have implicated gut microbiota in influencing response, as well as toxicity, across a range of treatments via a variety of mechanism.

Previous studies have detected gut microbiome features in lung cancer based on the histopathological features, however, these associations are less well characterized and require further investigation. Moreover, the mechanisms through which dysbiosis affects tumorigenesis and tumor growth across cancer types are numerous and varied. As we all know, the biomarkers of cytokeratin 19 fragment (CYFRA21-1), neuron specific enolase (NSE) and carcinoembryonic antigen (CEA) have the properties of high efficiency, convenience, easy access, low cost and smaller trauma. Therefore, these tumor biomarkers play important roles in the clinical application for early diagnosis, identification of the pathological type, tumor staging, monitoring of recurrence or metastasis, determination of efficacy and prediction of the prognosis of lung cancer 13. As we also know, CYFRA21-1 is mainly expressed in epithelial-derived cells thus the expression in lung squamous cell carcinoma (SCC) was higher than in lung adenocarcinoma and small cell lung carcinoma (SCLC). NSE has a high expression in SCLC by involving in energy metabolism, and is associated with TNM staging indicating a poor prognosis in SCLC 14. CEA is a broad-spectrum tumor marker which is useful for predicting recurrence and survival rates in many adenocarcinomas 15. In the light of the comprehensive mechanistic insights of biomarker, further preclinical studies need to solidify the relevance between gut microbiome dysbiosis and lung cancer, thus 16s sequencing analysis of the gut microbiome was performed to profile fecal samples in relation to lung cancer based on the expression of different biomarkers.

## Materials and Methods

### Samples

The fecal samples for 16S rRNA sequencing was obtained from 30 newly diagnosed lung cancer patients by histopathology and computed tomography (CT). The lung cancer patients were further divided into 3 groups based on biomarkers including CYFRA21-1 positive patients (CYF group, n=10), NSE positive patients (NSE group, n=9) and CEA positive patients (CEA group, n=11). No patients received chemotherapy, radiation therapy, targeted therapy, immunotherapy, or surgery for lung cancer before samples collection. Patients who had one of the following conditions were excluded: congestive cardiac failure, respiratory failure, renal failure, severe liver dysfunction, consumption of probiotics or antibiotics within 1 month before admission. The clinical characteristics of all participates are listed in Table 1 and the parameters of age and weight were comparable between each group (*P*>0.05). The control group was from 16 healthy participants who did not use any type of antibiotics or probiotics within 1 month before admission. For all participants, fresh fecal samples were collected into sterile EP tubes, and aliquots were frozen -80°C immediately until DNA extraction. Each patient has signed informed consent before the study. The protocol was approved by the ethics committee of the First Affiliated Hospital of Guangdong Pharmaceutical University.

### DNA extraction and amplification of theV4 Region of Bacterial 16S rRNA Genes sequencing

The microbial DNA was extracted from 46 fecal samples by the PowerSoil DNA Stool Mini Kit (MoBio) according to the manufacturer's recommended protocol. In brief, the V4 variable regions of the bacterial 16S rRNA gene were amplified by polymerase chain reaction using the universal primers 341F (CCTACGGGNGGCWGCAG) and 806R (GGACTACHVGGGTWTCTAAT). The extracted DNA was isolated by silica purification and quantified using a Mutiskan^TM^. The polymerase chain reaction cycle conditions were described previously: An initial denaturation at 95 °C for 2 minutes; followed by 30 cycles at 95 °C for 30 seconds, primer annealing at 52°C for 30 seconds, and extension at 72 °C for 45 seconds; and a final elongation at 72 °C for 5 minutes. PCR products were then visualized on 2% agarose gel. Subsequently, purified amplicons were pooled in equimolar amounts, and paired-end sequenced on Illumina HiSeq/MiniSeq for Genome Analysis.

### Microbiome Data Analysis

The raw FASTQ files were first de-multiplexed, then qualify-filtered using Chimera_check and merged using FLASH with the sequences were processed using the BIPES protocol.10 and QIIME 1.9 16, 17. Briefly, forward and reverse bacterial 16S rRNA reads were merged with a minimum merge length of 200bp, then simultaneously filtered to remove singletons and chimeras in UPARSE. Operational taxonomic units (OTUs) were defined basing on sequence similarity of 97%, and taxon-dependent analysis was assigned to individual OTUs through the Ribosomal Database Project (RDP) classifier using the Green Genes Database (HOMD) to explore lung cancer-associated differences in the fecal microbiota. The index of observed species, Chao1, Shannon and Simpson were used to calculate alpha diversity metrics. The beta diversity measurements including principal component analysis (PCA), principal co-ordinates analysis (PCoA) were analyzed by the unweighted UniFrac metric. The statistical significance was evaluated with analysis of similarities (ANOSIM). Pathway enrichment analysis was performed using Kyoto Encyclopedia of Genes and Genomes (KEGG) and database by PICRUSt 18, 19. Finally, the linear discriminant analysis (LDA) effect size (LEfSe) method was used to evaluate the influence of each differentially-abundant taxon.

### Statistical Analyses

Statistical tests were implemented using R (3.0.2; R Foundation for Statistical Computing) and Prism software (Graph Prism7.0 Software Inc. CA, USA). The data of age and weight are expressed as a mean ± standard deviation (SD) and the differences among the groups were evaluated by one-way analysis of variance (ANOVA). The Wilcoxon rank-sum test (for two groups) or Kruskal-Wallis test (for more than two groups) was used to analyse the diversity between groups comparisons. Fisher's exact test was performed on categorical variables. The chi-square test was used for categorical variables. A value of *P*< 0.05 was considered statistically significant in the compared groups.

## Results

### Taxonomic analysis of 16S rRNA V4 amplicon sequence data

**Figure 1:**
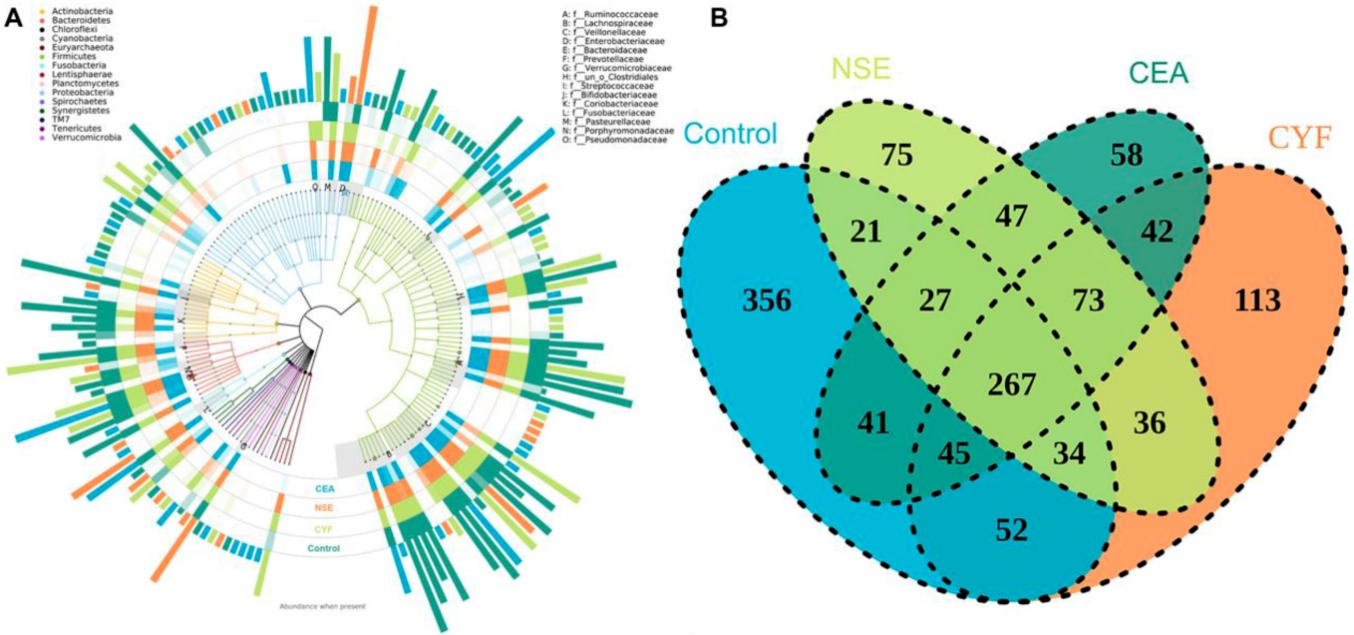
** (A)** The species tree and distribution of gut microbial community. **(B)** Venn diagrams shared OTUs between different groups.

**Figure 2:**
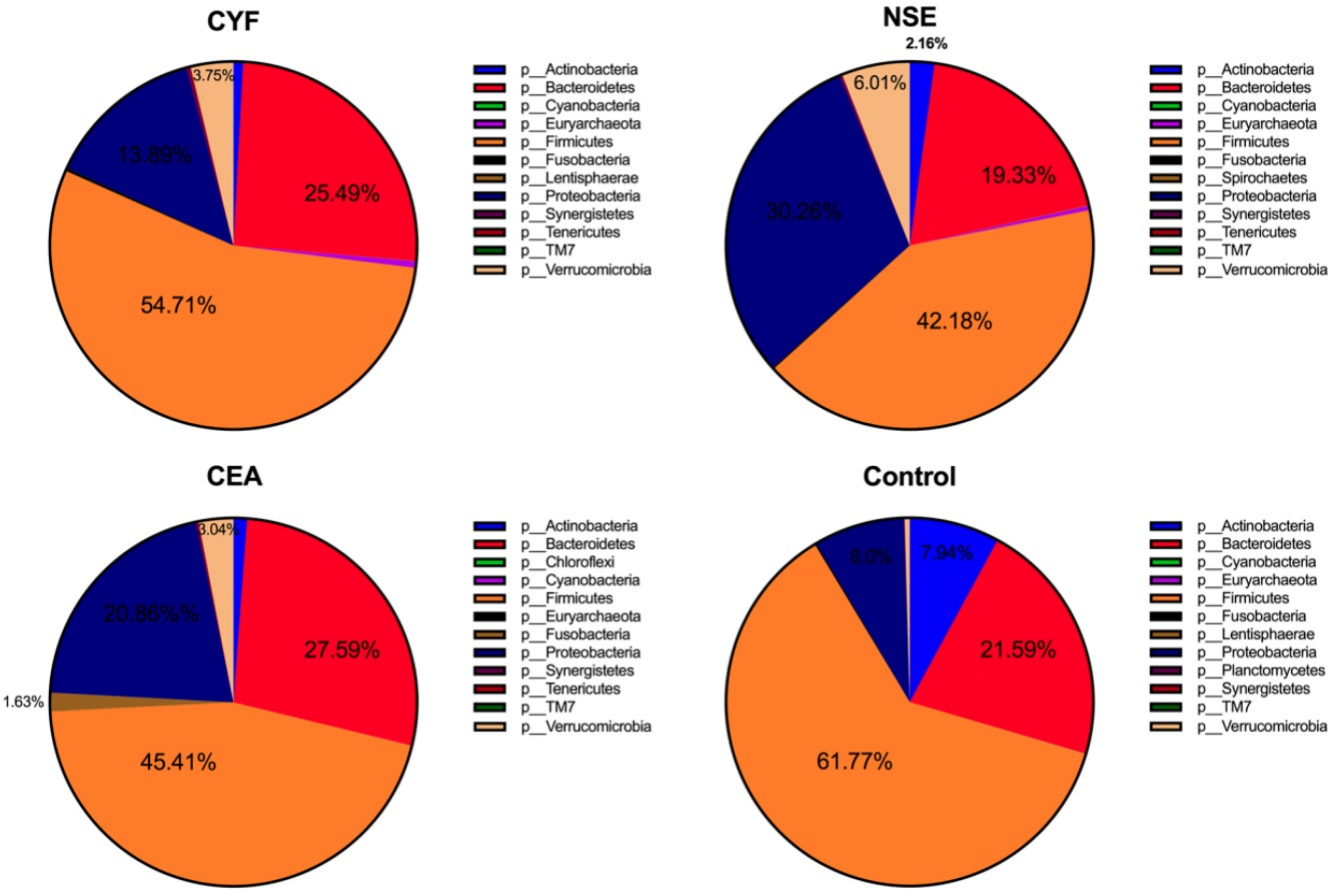
The taxonomic profile demonstrated the OTUs are assigned to prevalent microbiome of *Firmicutes, Bacteroidetes, Acidobacteria* and *Proteobacteria* at phylum level.

To explore the gut microbial community features of lung cancer patients, the microbiota relative taxon abundance in lung cancer groups were compared with healthy subjects. Total 1565 operational taxonomic units (OTUs) were annotated for subsequent analyses including 22 phyla, 116 family and 217 genera of gut microbes inferred from V4 amplicon sequencing ranging from 39 to 297 with 97% similarity among the samples (Figure 1A). The predominant genera were defined as the comprising greater than 1% of the total gut bacteria. The bacterial taxonomy distribution of three lung cancer groups demonstrated decreased density and clustering than healthy control. The venn diagrams reflecting the difference between each group as shown in Figure 1B, exhibited 113, 75, 58 and 356 in CYF, NSE, CEA and control group, respectively. At phylum level, *Firmicutes*, *Bacteroidete* and *Proteobacteria* were the most common phyla identified in three lung cancer groups, contributing 94.09% (CYF), 91.77% (NSE), 93.86% (CEA) of the gut bacteria, respectively. *Firmicutes*, *Bacteroidete, Proteobacteria* and *Acidobacteria* contributed to 99.3% of the gut bacteria in healthy control (Figure 2). The lung cancer groups had a conspicuously lower abundance in *Acidobacteria* and *Firmicutes*, relative higher abundance in *Proteobacteria* and *Verrucomicrobia* than the healthy control, especially in NSE group. The NSE group also showed a relatively low abundance of *Bacteriodetes*, while the CEA group showed more abundance of* Fusobacteria* (Figure 2). The ratio of two phyla (*Firmicutes* to *Bacteroidetes*) was decreased in lung cancer group 2.14 (CYF), 1.64 (CEA) and 2.18 (NSE).

**Figure 3:**
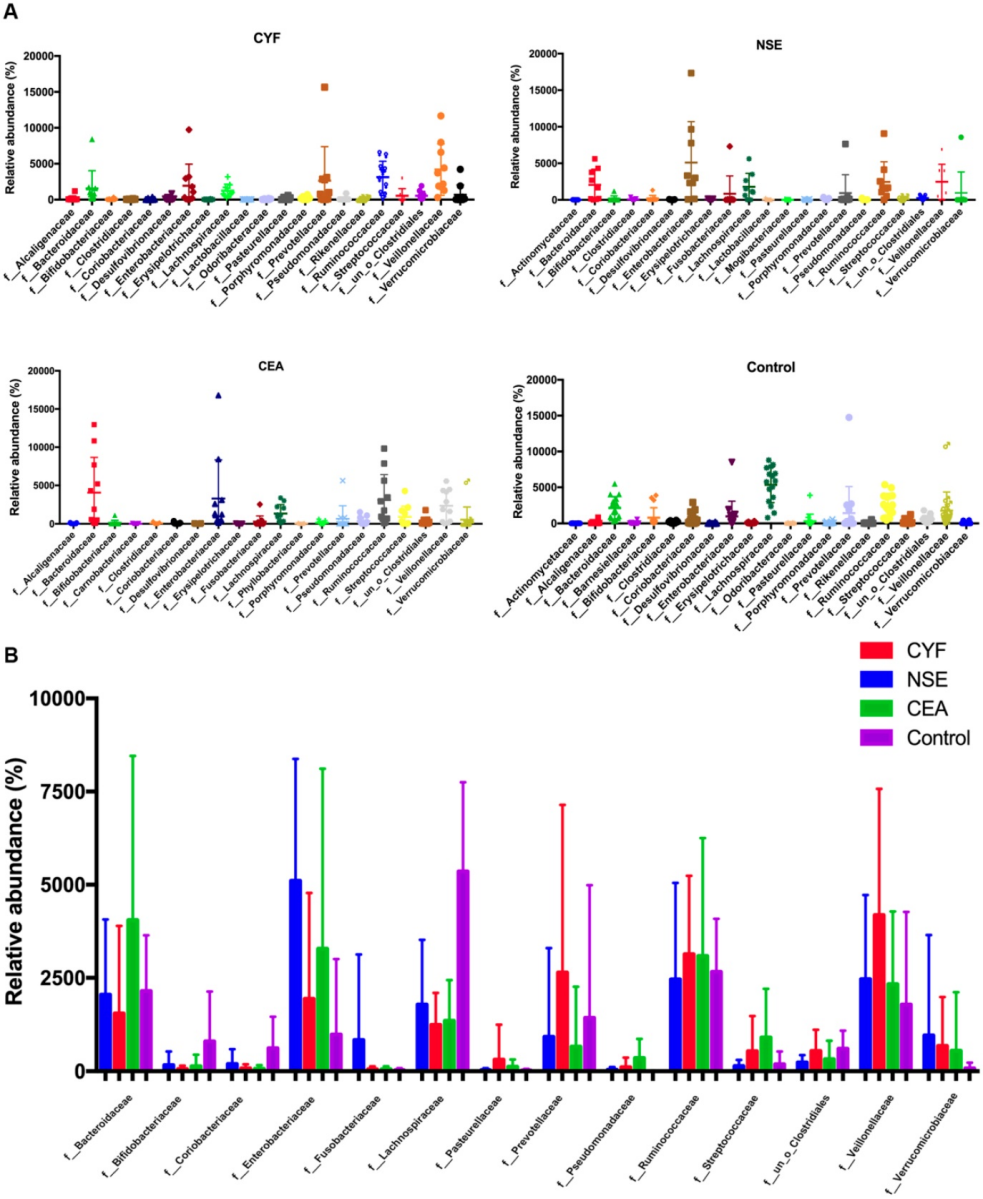
** (A)** Taxonomic summary of the gut microbiota of four groups at family level. **(B)** The comparison of relative abundant microbiome at family level between each group.

**Figure 4:**
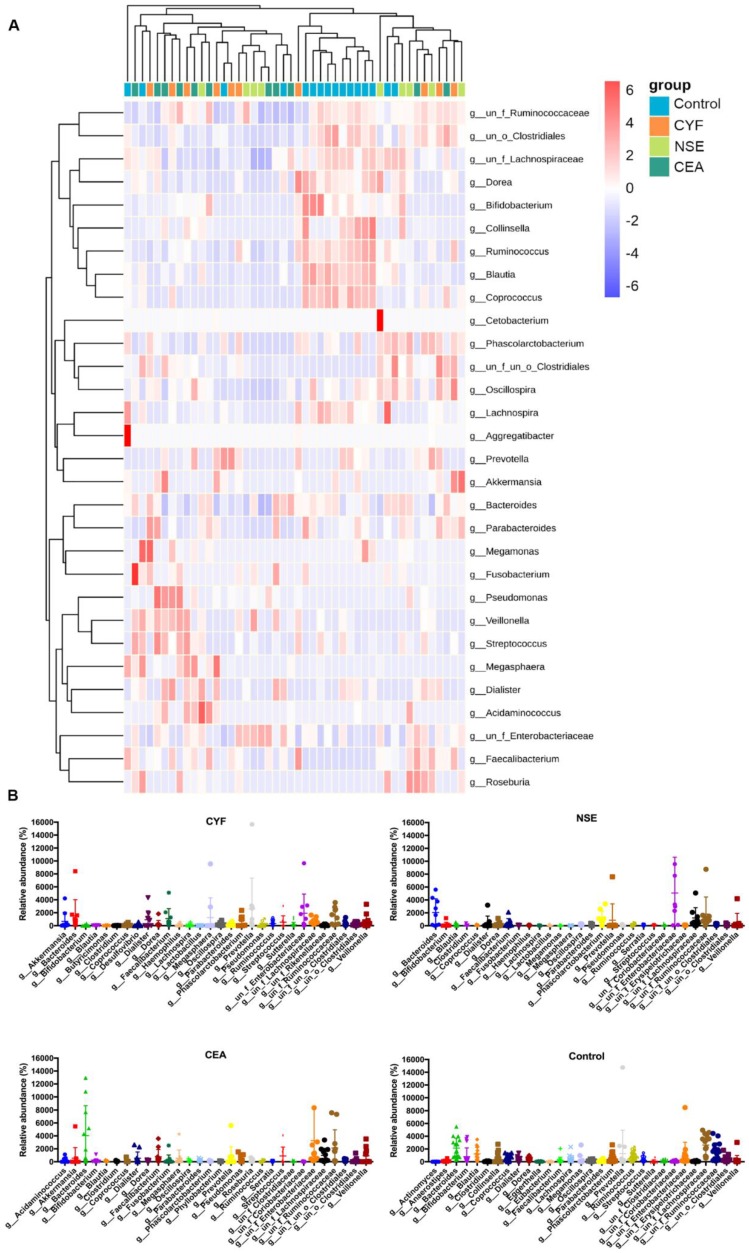
** (A)** LEfSe comparison of gut microbiota among CYF, NSE, CEA and Control groups. **(B)** Taxonomic summary of the gut microbiota of four groups at genera level.

At family level, the internal individual variation in taxonomic composition is higher and more dominant taxa are present in each group, especially in CYF and control group (Figure 3A). Among the total families identified in the gut bacterial, 49 in CYF group, 44 in NSE group, 40 in CEA group, and 47 in the control group of dominant family were detected. *Bacteroidaceae, Enterobacteriaceae*, *Lachnospiraceae, Prevotellaceae, Ruminococcaceae* and *Veillonellaceae* represented six most relative abundant microbiome in all groups (Figure 4). *Ruminococcus* is comparable in these four groups, while *Lachnospiraceae* is significant decreased in each lung cancer group*. Bacteroidaceae* and *Streptococcaceae* are relatively more abundant in CEA group than others. *Bacteroidaceae* is significantly different among CEA, CYF and healthy control group. *Enterobacteriaceae, Fusobacteriaceae* and *Verrucomicrobiaceae* are more abundant in NSE group than others, and significantly different between CYF and control group. *Prevotellaceae* and *Veillonellaceae* are more abundant in CYF group than others (Figure 3B).

The genera-level characterization is more complex, a number of bacterial genera were significantly differed between each group (Figure 4A). In CYF group, *Prevotella* (phylum *Bacteroidetes*)*, Enterobacteriaceae* (phylum* Proteobacteria*)*, Bacteroides* (phylum* Bacteroidetes*)*, Megasphaera* (phylum* Firmicutes*)*, Faecalibacterium* (phylum *Firmicutes*)*, Dialister* (phylum* Firmicutes*)*, Phascolarctobacterium* (phylum* Firmicutes*)*, Veillonella* (phylum* Firmicutes*)*, Akkermansia* (phylum* Verrucomicrobia*) and *Lachnospiraceae* (phylum* Firmicutes*) were the 10 dominant microbiota of 112 genera (Figure 4A). While, in NSE group, the dominant microbiota were *Enterobacteriaceae*, *Bacteroides*, *Ruminococcaceae* (phylum* Firmicutes*), *Lachnospiraceae*, *Phascolarctobacterium*, *Akkermansia*, *Prevotella*, *Cetobacterium* (phylum* Fusobacteria*), *Veillonella* and *Dialister* of 112 genera. The CEA group were significantly enriched for *Bacteroides, Enterobacteriaceae, Ruminococcaceae, Lachnospiraceae, Veillonella, Streptococcus* (phylum* Firmicutes*)*, Faecalibacterium, Prevotella, Megasphaera, Dialister* of 121 microbiome genera. While, *Lachnospiraceae, Bacteroides, Ruminococcaceae, Prevotella, Blautia* (phylum* Firmicutes*)*, Enterobacteriaceae, Bifidobacterium* (phylum *Actinobacteria*)*, Ruminococcus* (phylum* Firmicutes*)*, Coprococcus* (phylum* Firmicutes*) and *Phascolarctobacterium* were 10 main dominant microbiota of 131 genera in healthy control group (Figure 4B).

### The alpha diversity of the gut microbiota

**Figure 5:**
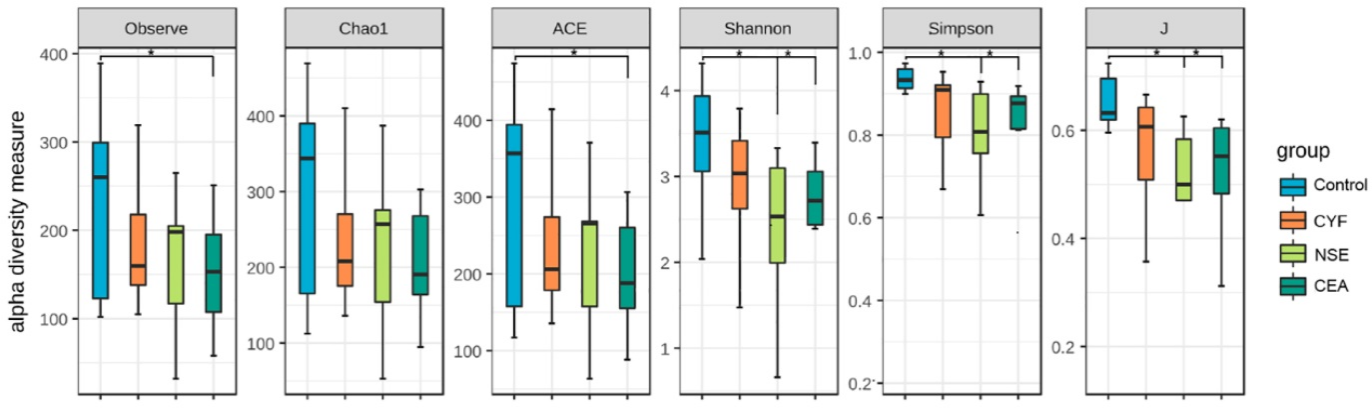
The comparison of gut microbiota alpha diversity between each group, including species richness (represented by Chao1, observed species) and evenness (represented by Shannon and Simpson index). Microbial community showed that the NSE and CEA group had less diversity, richness and evenness than the control group, whereas the CYF group showed a similar trend without significance compared to healthy control. Starred samples (*) were used to demonstrate the significant difference between the group.

To evaluate alteration in the microbiota community structure between each group, the microbial alpha diversity was measured as shown in Figure 5. The result indicates that alpha diversity of the gut microbiota in NSE (and CEA) groups was lower than that of healthy control in terms of Shannon and Simpson index (Shannon index -35.99* P*=0.0369, -31.16 *P*=0.0369; Simpson index 38.1944 *P* =0.0272, 33.11364 *P* =0.0386). The J index was also significantly different between NSE (or CEA) group and control (39.4028 *P*=0.0217 and 33.07955 *P*=0.0437, respectively) (Figure 2A). The richness index includes ACE and observed were no differences between each lung cancer group and healthy control group.

### The β-diversity analysis

**Figure 6:**
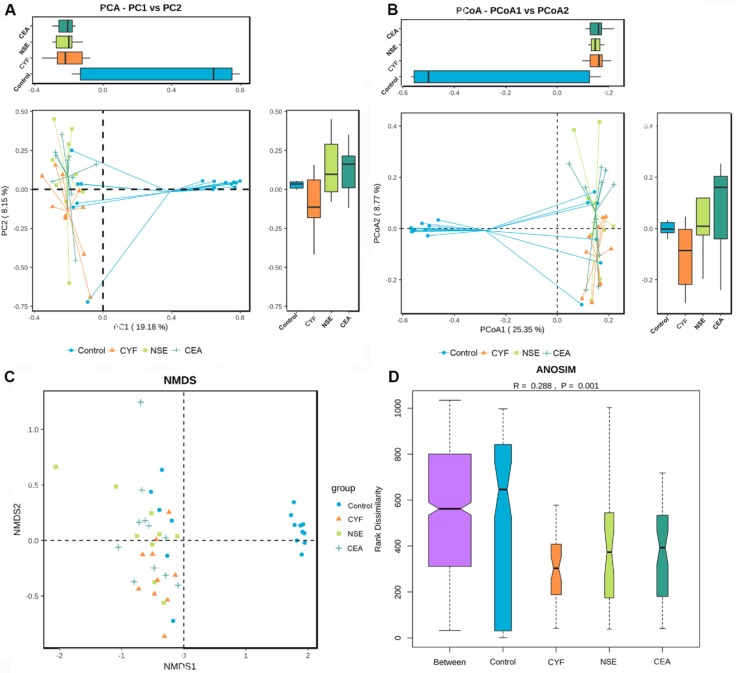
** (A)** Principal coordinate analysis, Principle coordinate analysis, **(B)** Non-metric multi-dimensional scaling **(C)** and Analysis of similarities **(D)** illustrating the grouping patterns of the CYF, NSE, CEA and control groups based on the Bray-Curtis and Unweighted UniFrac Distances. Each dot represents a sample, and the corresponding group can be found in the legend. Distances between any pair of samples represent the dissimilarities between each sample. There was significant difference in β-diversity between the four groups.

The principal component analysis (PCA) and principal co-ordinates analysis (PCoA) based on unweighted uniFrac distance (Figure 6A) and weighted uniFrac distance (Figure 6B) indicated the stool microbiome of lung cancer patients clustered significantly separately from that of healthy controls. Non-metric multi-dimensional scaling (NMDS) analysis based on Bray-Curtis distances between microbial genera revealed significant differences between lung patients and healthy controls (Figure 6C). The analysis of similarities (ANOSIM) indicated that the structure of the gut microbiota significantly differed between each group (ANOSIM, r = 0.288, *P* = 0.001) (Figure 6D).

### Functional profile of the gut microbiome

**Figure 7:**
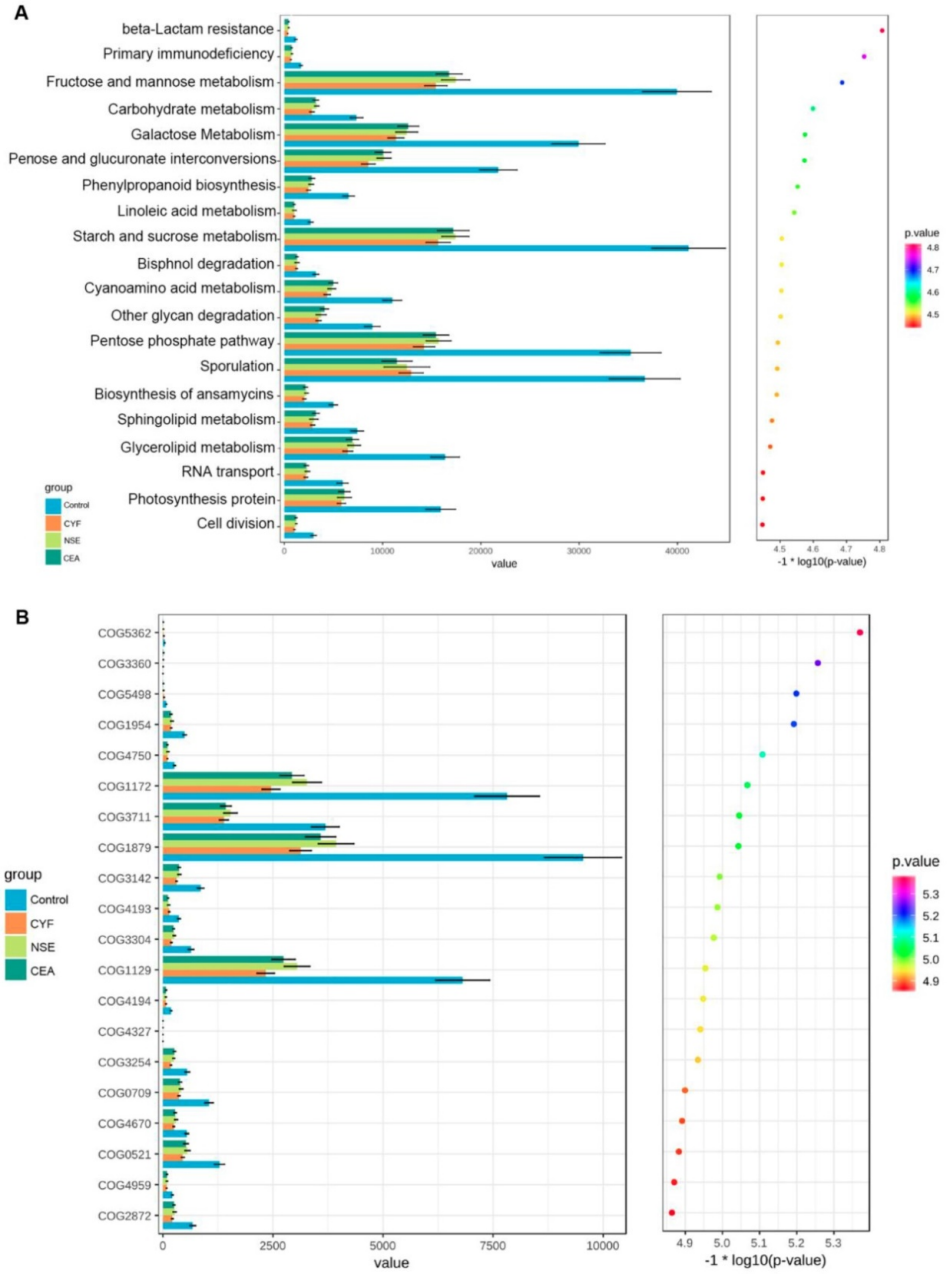
** (A)** KEGG pathway was less abundant in lung cancer groups than healthy control. **(B)** COG pathway was less abundant in lung cancer groups than healthy control.

KEGG and COG pathway comparisons were performed to explore potential differences in the functional composition of the microbiome of lung cancer patients versus controls. Although the functional composition of lung cancer patients and controls was highly similar, the microbiome of lung cancer patients showed lower abundance in pathways of energy metabolism and ABC-type transport than the healthy controls. The analysis showed the clustering of metabolic modules including fructose and mannose metabolism, galactose metabolism, pentose and glucuronate interconversions, starch and sucrose metabolism, pentose phosphate pathway and sporulation (Figure 7A). In addition, periplasmic component (COG1879), ATPase component (COG 1129) and permease components (COG1172) of ABC-type transport system were significantly less abundant in lung cancers than healthy control, which promote utilization of glucose, ribose/galactoside to regulate the energy. Likewise, Transcriptional antiterminator (COG3711) orthologue was also less abundant lung cancers than healthy control (*p*<0.05) (Figure 7B).

## Discussion

This is the first study of human gut microbiome 16s RNA sequencing in lung cancer patients based on biomarker, which was different from the traditional pathological classification of cancer 4, 20. As all known, NSE is expressed in SCLC, while CYFRA 21-1 is frequently expressed in squamous lung cancer. CEA is a tumor marker that is useful for predicting recurrence in many adenocarcinomas 15. In the light of the multiple functions biomarkers play in the diagnosis and prognosis in lung cancer, the possible relationship between gut microbiome and lung cancer needs to be explored. The results indicated that all lung cancer groups demonstrated an elimination, low-density, and loss of bacterial diversity microbial ecosystem compared to that of the healthy control.

At phylum level, the gut microbiome samples show lower abundance in *Firmicutes* and *Actinobacteria* in all lung cancer groups compared to that of control group. As all known, all butyrate-producing bacteria belongs to *Firmicutes* phylum in human bacterial communities. While, butyrate is one of the most crucial fatty acids and is associated with anti-inflammatory activities, cellular proliferation, inducing differentiation of regulatory T cells and apoptosis by activating signal pathways 1, 21. *Actinobacteria* is valeric acid-associated bacteria, which was also found abundant in autistic individuals 22. Meanwhile, lung cancer groups also show lower ratio of *Firmicutes* to *Bacteroidetes* than healthy control group. The high ratio of *Firmicutes*/*Bacteroidetes* is often seen in healthy adults as exemplified in one large gut microbiome cohort study on 1135 Dutch healthy participants 23. Reduced *Firmicutes*/*Bacteroidetes* ratio has been repeatedly reported associated with the dysbiosis of gastrointestinal tract metabolism, which resulted in a low concentration of circulating short-chain fatty acids, and then influenced elements for host systemic immunity and systemic inflammation 22. These evidences suggest a disruption of the equilibrium in gut microbiota of lung cancer patients and presence of different microbiome features from chronic respiratory disease such as asthma, chronic obstructive pulmonary disease (COPD) which exhibit relative enriched microbiomes with phyla. Moreover, chronic gastrointestinal tract (GIT) diseases, such as inflammatory bowel disease (IBD) or irritable bowel syndrome (IBS) were notified often occur together with chronic lung diseases 24. As a result, gut-lung axis was proposed in the pathogenesis in the lung and gut diseases 25.

We also noticed that the microbiome of lung cancer groups demonstrates relative higher abundance of *Proteobacteria* and *Verrucomicrobia* than healthy control, especially in NSE group. NSE is highly expressed in SCLC by being involved in energy metabolism and associates with TNM staging indicating a poor prognosis in patients with SCLC. *Proteobacteria* is an opportunistic pathogen, constituting a major structural imbalance of gut microbiota in lung cancer patients. However, *Verrucomicrobia* phylum is occasionally observed in healthy and was reported to stand out for its richness in Chilean subjects 26. Moreover, the NSE group demonstrated low abundance of *Bacteroidetes*, some genus of which is essential for the host by performing metabolic conversion such as degradation of proteins or complex sugar polymers. Interestingly, the CEA group has relative higher level of *Fusobacteria*, which is reported to be correlated with the development of several types of malignant tumor 27. *Fusobacteria* is a potential inducer of T regulatory cells or carcinogens, promotes autophagy activation with poor outcomes in malignancies such as colon cancer. These results indicate a potential link between gut bacteria and tumor biomarker within lung cancer. We suspected that the tumor cells might create metabolic products and exhibit differential profiles which may favor the selective adherence of some members of the microflora.

The characterizations in family and genera levels are more complex and significantly varied from each group, presenting more different pathogen microbiome and well-characterized structures. The probiotic bacteria of *Bacteroides* that belong to *Bacteroidaceae* family are of lower abundance in each lung cancer group than healthy control. The probiotic* Bacteroides* genus have been previously reported to enhance anti-CTLA4 immune checkpoint efficacy in mice and are presumed to directly contact and stimulate host DCs and T cells via pathogen-associated molecular patterns 28. The genus *Bifidobacterium* is lacking in all lung cancer groups which is considered as an important component of the commensal microbiota and plays important roles in several homeostatic functions such as immune, neurohormone and metabolism 29, 30. The pathogen *Prevotella* presents higher proportions in cancer patients especially in CYF group, while *Streptococcus* is only seen in CEA group. *Prevotella* belonging to *Prevotellaceae* family was reported to be linked to chronic inflammatory conditions, such as arthritis, mucosal and systemic T-cell activation in untreated human immunodeficiency virus infection 31. Some species of *Streptococcus* genus can cause unique abscesses and are linked to colorectal cancer 32. *Enterobacteriaceae*, as a significantly opportunistic pathogen, exists in the human gut without causing symptoms or diseases under normal conditions. However, a significant variation of *Enterobacteriaceae* may be caused by host immunity and environmental factors such as redox state and oxygen availability 33. *Ruminococaceae* and *Lachnospiraceae* genera both belong to *Firmicutes* phylum and were significant decrease in each lung cancer group*. Ruminococaceae* has been reported to be associated with both response and toxicity to immune checkpoint blockade.* Lachnospiraceae* can protect host against cancer by producing butyric acid 34. *Dialister* is detectable in all lung cancer groups and belongs to the *Veillonellaceae* phylum. The succinate-utilizing *Phascolarctobacterium* is lacking in CEA group, which is associated with lower concentrations of lipopolysaccharide-binding protein and C-reactive protein 35. The mucus-degrading genera *Akkermansia* detected in CYF and NSE groups is the only identified member of *Verrucomicrobia* phylum, which is proposed as a hallmark of healthy gut status due to its anti-inflammatory and immunostimulant properties and its ability to improve gut barrier function, insulin sensitivity and endotoxinemia 26. It has been proposed that the growth of this bacterium is favored by low availability of enteral nutrients such as in long-term fasting and malnutrition. *Megasphaera* is a genus of *Firmicutes*, which was only seen in CYF and CEA groups. Meanwhile, CYF and CEA groups both have a relative higher abundance of health-promoting bacteria of *Faecalibacterium* genus, acting in synergy with anti-PD1 in cancer treatment. *Faecalibacterium* is also known as a potent SCFA producer and the proportion could be altered by high-calorie diets, while *Phascolarctobacterium* is main dominant microbiome in NSE group. Each lung cancer group exhibited decreased abundance of *Blautia* genus belonging to *Firmicutes* phylum, which has a role of helping digest complex carbohydrates. The decreased of* Blautia* genus was also seen in irritable bowel syndrome, nonalcoholic fatty liver diseases, Crohn's disease and diabetes. The level of *Veillonella* genus is higher in each lung cancer group, which is well known for its lactate fermenting abilities and recently was reported to be diagnostic marker for both SCC and adenocarcinoma. *Coprococcus* is only seen in control group which is beneficial butyrate-producing genus. These results indicate that the composition and development of bacterial communities varies in lung cancer with different biomarkers. Therefore, it is possible that some special microbiome may serve as a diagnosis, prognosis, therapeutic target or fecal microbiota transplantation in lung cancer therapy. Moreover, it is debatable now whether cancer is the product of variations in the microbiota, or whether modifications in the normal microbiome are the result of cancer progression.

The KEGG and COG pathway analysis indicated that the dysbiosis of gut bacteria in lung cancer is strongly associated with dysregulation of basic metabolic processes such as energy metabolism, transport and sporulation 18. It shows that carbohydrate transport and metabolism has less abundant functional group in gut microbiome of lung cancer groups. Energy metabolism in the cancer system has been suggested to be an important factor contributing to the pathogenesis of cancer, while the gut microbiota is vital for the development of the immune system and homeostasis. These bacteria may shed different microbial bioactive molecules and affect the host including fructose and mannose metabolism, galactose metabolism, pentose and glucuronate interconversions, starch and sucrose metabolism, pentose phosphate pathway and sporulation. *Firmicutes* plays an important role in this process, which could transform the undigested carbohydrates and proteins into acetic acid, and then produce energy for organism. Moreover, the decreased abundance in the ABC-type transport system signaling pathways further suggest potential energic and metabolic alteration of these gut microbiota in lung cancer. Therefore, there are complex interactions between gut microbiota and the host, and as yet, our knowledge about these comprehensive interactions is limited. This observation is compatible with the hypothesis that lung cancer is fundamentally a metabolic disease, and patients with lung cancer often display a coexisting metabolic disorder phenotype in conjunction with the pathology. It is reported that microbiota-produced butyrate could provide energy to colonocytes and then prevent autophagy in the gut. In addition, physiological homeostasis may be disrupted by the microbiota, resulting in disruption of host metabolism, immune dysregulation, neurological and cognitive dysfunction and others. Therefore, we speculated that gut microbiota affects the host via the immune system or metabolites, which provides an improved understanding of perturbations of the microbiome-metabolome interface in lung cancer and may identify some potential diagnostic and therapeutic targets. However, it is not necessarily that the functions of the microbiota are solely dependent on any one of these interactions, and alterations in these relationships may affect lung cancer system and cause subtype. This process was hypothesized to be induced by the production of carcinogenic metabolic products or by the production of toxins via impairing immune responses as well as modulating the inflammatory response of the host.

Emerging evidence indicates interventions in microbiome could improve anti-cancer therapy efficacy as well as to ameliorate chemotherapy-related toxicity, as gut microbiome performs a number of vital functions in vitamin production, dietary compound metabolization, protection against gut pathogen expansion and systemic infiltration 36. Therefore, some probiotics are prescribed to fight dysbiosis in cancer patients subjected to chemotherapy and radiotherapy. As all known biomarkers not only have prognostic values, but also can help guild treatment decisions. Over the last decade, tissue and/or blood biomarkers have become a helpful guider in the treatment decisions of patients with advanced NSCLC, because increasing evidence shows that lung cancer patients with targeted therapies have superior clinical outcomes compared to those with traditional chemotherapy 37. The association of gut microbiome and biomarkers in lung cancer present in this study may provide new inspiration for the study of possible roles of biomarkers played in lung cancer system and its influence on chemotherapy. Therefore, a more rigorous randomized controlled trial should be designed to elaborate on this important topic.

In summary, this is the first study that comparatively evaluated gut microbiota features in lung cancer based on different tumor biomarkers, providing new insight into the association between dysbiosis gut microbiota and lung cancer and possible links to biomarkers. The composition of the microbiota varied in each group, which demonstrated low density microbiome features in lung cancer. Meanwhile, both the COG and KEGG pathways analyses indicated decreased abundance of some dominant metabolism-related pathways in lung cancer. These evidences indicated dysbiosis of gut bacteria in lung cancer is associated with dysregulation of basic metabolic and immunologic functions contributing to the development and differentiation of the lung cancer system. However, we cannot rule out that the altered bacteria diversity may be a passive by-product of tumor progression. Meanwhile, the method of 16S rRNA gene sequencing has known limitations in determining the microbiota components, such as the potential for skewing the results owing to amplification bias and inability to identify most microbes at the species level. Therefore, large-scaled studies and metabolic analyses need to further validate microbial biomarkers for different type histopathological lung cancer patients.

## Figures and Tables

**Table 1:** Descriptive data of included subjects in the study

Groups(n)	Gender(M/F)	Age		Weight	
		Mean ± SD	*P* value	Mean ± SD	*P* value
CYFRA (n=10)	7/3	60.4 ± 12.2	*F=0*.510 *P*>0.05	56.45 ± 8.42	*F* = 0.711 *P*>0.05
CEA (n=11)	6/5	56.82 ± 10.08		58.95 ± 10.42	
NSE (n=9)	8/1	62.39 ± 9.20		56.67 ± 10.43	
Control (n=16)	9/7	59.12 ± 7.76		60.44 ± 8.03	
